# Development of a multiplex RT-PCR assay for simultaneous detection of *Lily symptomless virus*, *Lily mottle virus*, *Cucumber mosaic virus*, and *Plantago asiatica mosaic virus* in Lilies

**DOI:** 10.1186/s12985-022-01947-3

**Published:** 2022-12-16

**Authors:** Leifeng Xu, Jun Ming

**Affiliations:** Institute of Vegetables and Flowers, Chinese Academy of Agricultural Sciences, Beijing, 100081 China

**Keywords:** *Lilies*, Multiplex RT-PCR, LSV, LMoV, CMV, PlAMV

## Abstract

**Background:**

Viral pathogens causing significant economic losses in lilies (*Lilium* spp. and hybrids) include *Lily symptomless virus* (LSV), *Lily mottle virus* (LMoV), *Cucumber mosaic virus* (CMV), and *Plantago asiatica mosaic virus* (PlAMV). Rapid and efficient virus detection methods are pivotal to prevent the spread of these viruses.

**Results:**

In this study, four specific primer pairs designed from conserved regions of genomic sequences of each virus were used to amplify a 116 bp product for LSV, a 247 bp product for LMoV, a 359 bp product for CMV, and a 525 bp product for PlAMV in a multiplex reverse transcription-polymerase chain reaction (multiplex RT-PCR). The amplified products were clearly separated by 2% agarose gel electrophoresis. The optimal reaction annealing temperature and cycle number were 53.8 °C and 35, respectively. The developed multiplex RT-PCR method was then used to test virus infections from lily samples collected from different regions of China.

**Conclusions:**

An effective multiplex RT-PCR assay was established for the simultaneous detection and differentiation of LSV, LMoV, CMV, and PlAMV in lilies, which offers a useful tool for routine molecular diagnosis and epidemiological studies of these viruses.

**Supplementary Information:**

The online version contains supplementary material available at 10.1186/s12985-022-01947-3.

## Background

Lilies are one the most important flower bulb plants, and widely used as cut flowers, flowering potted plants, and landscape plants. Besides, some lily species are cultivated as vegetables and medicines [1]. Viral diseases are one of main limiting factors in lily cultivation. More than 10 different viruses have been reported to infect lilies [2]. Among them, *Lily symptomless virus* (LSV; genus *Carlavirus*), *Cucumber mosaic virus* (CMV; genus *Cucumovirus*), *Lily mottle virus* (LMoV; genus *Potyvirus*), and *Plantago asiatica mosaic virus* (PlAMV; genus *Potexvirus*) are four of the most prevalent economically important viruses [3–5]. LSV-infected lilies exhibit symptoms of leaf mottle, vein clearing, and diminished growth [3]. LMoV often cause flower-color breaking, leaf mottle, leaf mosaic, chlorotic and yellow streaking, vein clearing, leaf curling, and narrowing [4]. CMV-infected lilies exhibit typical symptoms of brittleness of leaves and malformation of flowers and flower buds [4]. PlAMV-infected lily leaves show rust-brown necrotic spots and are often brittle [5].These viruses can be transmitted from generation to generation through clonal propagation, which is the major propagation method used for the commercial production of lily bulbs. Single and multiple viral infections with these viruses cause a significant reduction in bulb quality and yields and reduce the value of lily flowers. To prevent the spread of these viruses, a rapid, reliable, and specific virus detection method is urgently needed for the simultaneous detection of these viruses.

Currently, several techniques have been developed to detect viruses [6–8]. ELISA, simplex RT-PCR, uniplex real time RT-PCR, and LAMP have been developed for the detection of lily viruses [4, 9–11]. Multiplex RT-PCR amplifying multiple nucleic acid fragments in one reaction enables rapid and sensitive identification of several viruses simultaneously in a single assay, which greatly reduces cost and increases efficiency of viral surveys [12, 13]. To date, multiplex RT-PCR has been used widely to detect viruses in chrysanthemum, soybean, maize, pepper, apple, and tomato [14–20].

In this study, four specific primer pairs that can amplify DNA fragments of different sizes were designed according to genomic sequences of LSV, LMoV, CMV, and PlAMV. After optimizing RT-PCR conditions, an efficient multiplex RT-PCR assay was established and validated forthe simultaneous of LSV, LMoV, CMV, and PlAMV infecting lily plants.

## Methods

### Plant materials

Fresh leaves from *Lilium* ‘Tiny Padhye’ grown in greenhouses at the Chinese Academy of Agricultural Sciences (Beijing, China), which were confirmed to be co-infected with LSV, LMoV, CMV, and PlAMV by RT-PCR and sequencing were used to set up and optimize the multiplex RT-PCR assay.

### RNA extraction and reverse transcription

Total RNA was isolated from the lily leaf samples as described before [21]. First strand cDNA was synthesized from 5 µg total RNA using a SuperScript III reverse transcription kit (Invitrogen, Carlsbad, CA, USA) in a 20 µL reaction mixture with random primers, according to the manufacturer’s protocol.

### Design of virus-specific primers

The genomic sequences of LSV (accession numbers: KT923149.1, KJ627242.1, LC720794.1, LC704532.1, KC884548.1, KF958301.1, FJ456352.1, DQ531052.1, KJ627237.1, and LC649240.1), LMoV (accession numbers: MK368809.1, MK368792.1, MF983709.1, MT784663.1, KJ627230.1, JQ710690.1, AB054886.1, AJ310203.1, and LC733900.1), CMV (accession numbers: MN057671.1, HG316104.1, JN830618.1, EF158110.1, AY374328.1, AJ495841.1, LC733907.1, AM040193.1, and AJ131615.1), and PlAMV (accession numbers: KX245539.1, LN794199.1, KF601695.1, KU159091.1, LC422371.1, KU159089.1, LC592411.1, and KU159093.1) were obtained from GenBank. Nucleotide sequence alignment was performed using ClustalW implemented in MEGA X [22], and conserved regions of each virus were identified. Virus-specific primers were designed based on the identified conserved regions of each virus using Primer Premier 5.0 software (Premier Bio-soft International, Palo Alto, CA) (Additional file 1: Fig. S1–S4, Table 1).

**Table 1:** Primers for uniplex RT-PCR assay and multiplex RT-PCR assay

Virus	Primer	Primer sequence (5′–3′)	Products	Target gene
LSV	LSV-F	TGGCAACCACTGAACAAA	116 bp (OP680588)	CP
	LSV-R	GCTCACGCAAGCACACAT		
LMoV	LMoV-F	CGCATTTCTCACATCTCGC	247 bp (OP680591)	CP
	LMoV-R	TCTTGGGTGGTCACCTTTC		
CMV	CMV-F	CACATCTATCACCCTAAAGC	359 bp (OP680590)	CP
	CMV-R	TTGAATACACGAGGACGG		
PlAMV	PlAMV-F	ACCAACTCTGGGCCTTACA	525 bp (OP680589)	RdRp
	PlAMV-R	TCTGGGTGCTTTCCGTCA		

### Evaluation of primer specificity

Uniplex RT-PCR assays were performed to evaluate the specificity of primer pairs using a multi PCR Kit (Tiangen, Beijing, China). Each 50 μL reaction mixture contained 2.5 μL each primer (2 μM), 2 μL cDNA, 4 μL super pure dNTPs (2.5 mM each), 5 μL 10 × multi hotstart buffer, 1 μL multi hotstart DNA polymerase, and 33 μL double-distilled water. The thermal cycling conditions were as follows: 95 °C for 15 min, followed by 35 cycles of 94 °C for 30 s, 55 °C for 60 s, 72 °C for 90 s, and a final extension at 72 °C for 10 min. The PCR products were electrophoresed in a 2% agarose gel in 0.5 × TBE buffer.

To further confirm primer specificity, PCR products were cloned into the pEasy-T1 vector (Transgen, Beijing, China) for sequencing. Sequence alignment was performed using DNAMAN 5.0 Software (Lynnon Biosoft, San Ramon, CA, USA).

### Optimization of multiplex RT-PCR assay

The multiplex RT-PCR was optimized by varying the cycling conditions (annealing temperature and the number of amplification cycles). Different annealing temperatures were set from 53.0 to 65.0 °C (53.0 °C; 53.8 °C; 55.3 °C; 57.6 °C; 60.3 °C; 62.6 °C; 64.1 °C; 65.0 °C). The number of amplification cycles ranged from 25 to 40 (25; 30; 35; 40). The PCR products were analyzed as described above.

### Sensitivity of multiplex PCR assay

To compare the sensitivity of multiplex RT-PCR versus, 10-fold serial dilutions of cDNA from lily samples co-infected with LSV, LMoV, CMV, and PlAMV were used as templates. The PCR products were analyzed as described above.

### Survey of lily viruses by multiplex RT-PCR assay

A total of 180 lily samples were collected from different regions of China for the detection of LSV, LMoV, CMV, and PlAMV using the developed multiplex RT-PCR assay. Of them, 30 samples were an edible lily *(Lilium lancifolium* Thunb.) collected from Xiangxi, Hunan province; 30 samples were an edible lily (*Lilium davidii* var. unicolor) collected from Lanzhou, Gansu province; and 120 samples were ornamental lily cultivars collected from other regions (Table 2).

**Table 2:** Detection of four viruses in lily plants from different geographic regions of China using multiplex RT-PCR assay

Location	No. of samples	No. of positive samples and positive rate (%)			
		LSV	LMoV	CMV	PlAMV
Xiangxi, Hunan province	30	10 (33.33)	27 (90.00)	18 (60.00)	2 (6.67)
Lanzhou, Gansu province	30	30 (100.00)	1 (3.33)	5 (16.67)	0 (0.00)
Kunming, Yunnan province	30	7 (23.33)	11 (36.67)	12 (40.00)	4 (13.33)
Xinyi, Guizhou province	30	7 (23.33)	8 (26.67)	25 (83.33)	3 (10.00)
Beijing	30	9 (30.00)	10 (33.33)	20 (66.67)	4 (13.33)
Lianyungan, Jiangsu province	30	7 (23.33)	10 (33.33)	17 (56.67)	3 (10.00)
Total	180	70 (38.89)	67 (37.22)	97 (53.89)	16 (8.89)

## Results

### Specificity and compatibility of primer pairs

**Fig. 1:**
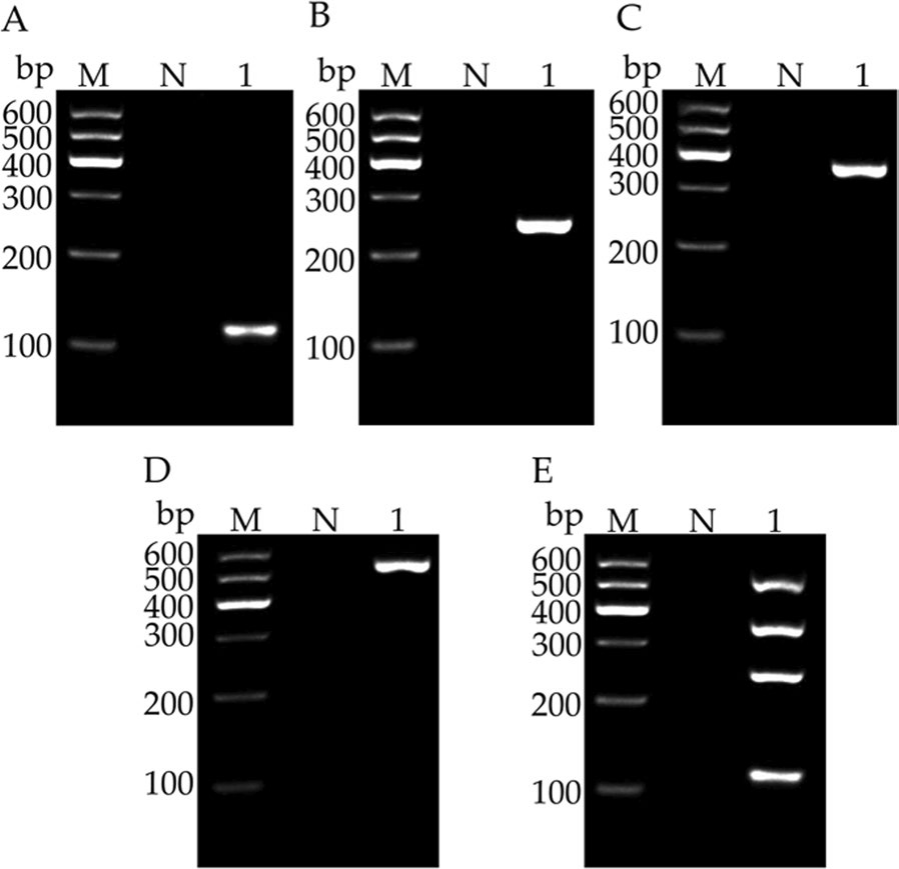
Determination of specificity and compatibility of four primer pairs used for uniplex RT-PCR to detect LSV (**A**), LMoV (**B**), CMV (**C**), and PlAMV (**D**) and for multiplex RT-PCR assay (**E**). Lane M, 100 bp plus DNA ladder; Lane N, negative control

In the uniplex RT-PCR, fragments of the expected size for LSV, LMoV, CMV, and PlAMV were amplified specifically using virus-specific primer pairs (Fig. 1A–D). In multiplex RT-PCR, mixed primers could also amplify fragments of the expected size for the four viruses with no non-specific bands (Fig. 1E). To further confirm the specificity and compatibility of the designed primer pairs, the amplified fragments were cloned separately and sequenced. Sequencing analysis showed that the sequences of the amplified products were highly homologous to the corresponding sequences of LSV, LMoV, CMV, and PlAMV (data not shown). These results showed that the primers could be used to separate the four lily viruses in the developed multiplex RT-PCR assay.

### Optimization of multiplex RT-PCR assay

**Fig. 2:**
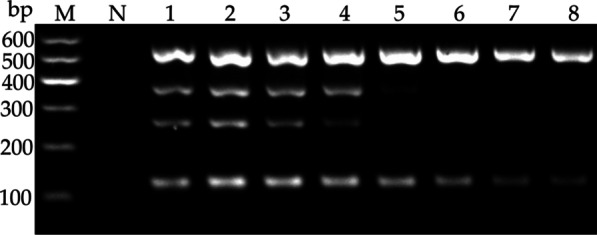
Optimization of annealing temperature for multiplex RT-PCR assay to detect LSV, LMoV, CMV, and PlAMV. Lane M, 100 bp plus DNA ladder; Lane N, negative control; Lane 1–8, 53.0 °C; 53.8 °C; 55.3 °C; 57.6 °C; 60.3 °C; 62.6 °C; 64.1 °C; 65.0 °C

**Fig. 3:**
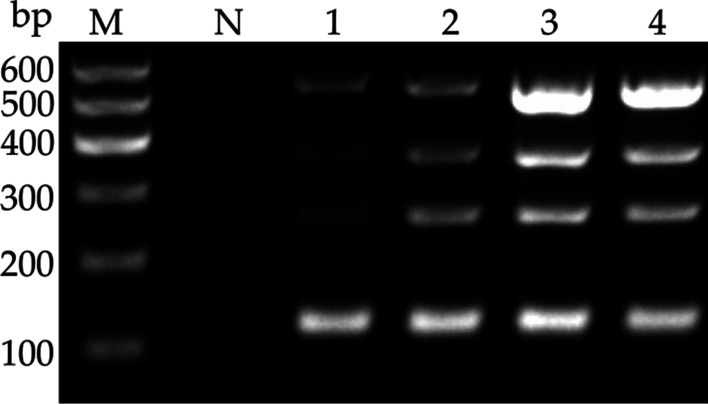
Optimization of amplification cycle number for multiplex RT-PCR assay to detect LSV, LMoV, CMV, and PlAMV. Lane M, 100 bp plus DNA ladder; Lane N, negative control. Lanes 1–4, 25 cycles; 30 cycles; 35 cycles; 40 cycles

The multiplex RT-PCR was optimized by varying the annealing temperature and the number of amplification cycles. According to the amplification efficiency and specificity of the virus-specific primers, the optimal annealing temperature and number of cycles were determined to be 53.8 °C and 35, respectively (Figs. 2, 3).

### Sensitivities of uniplex RT-PCR and multiplex RT-PCR assays

**Fig. 4:**
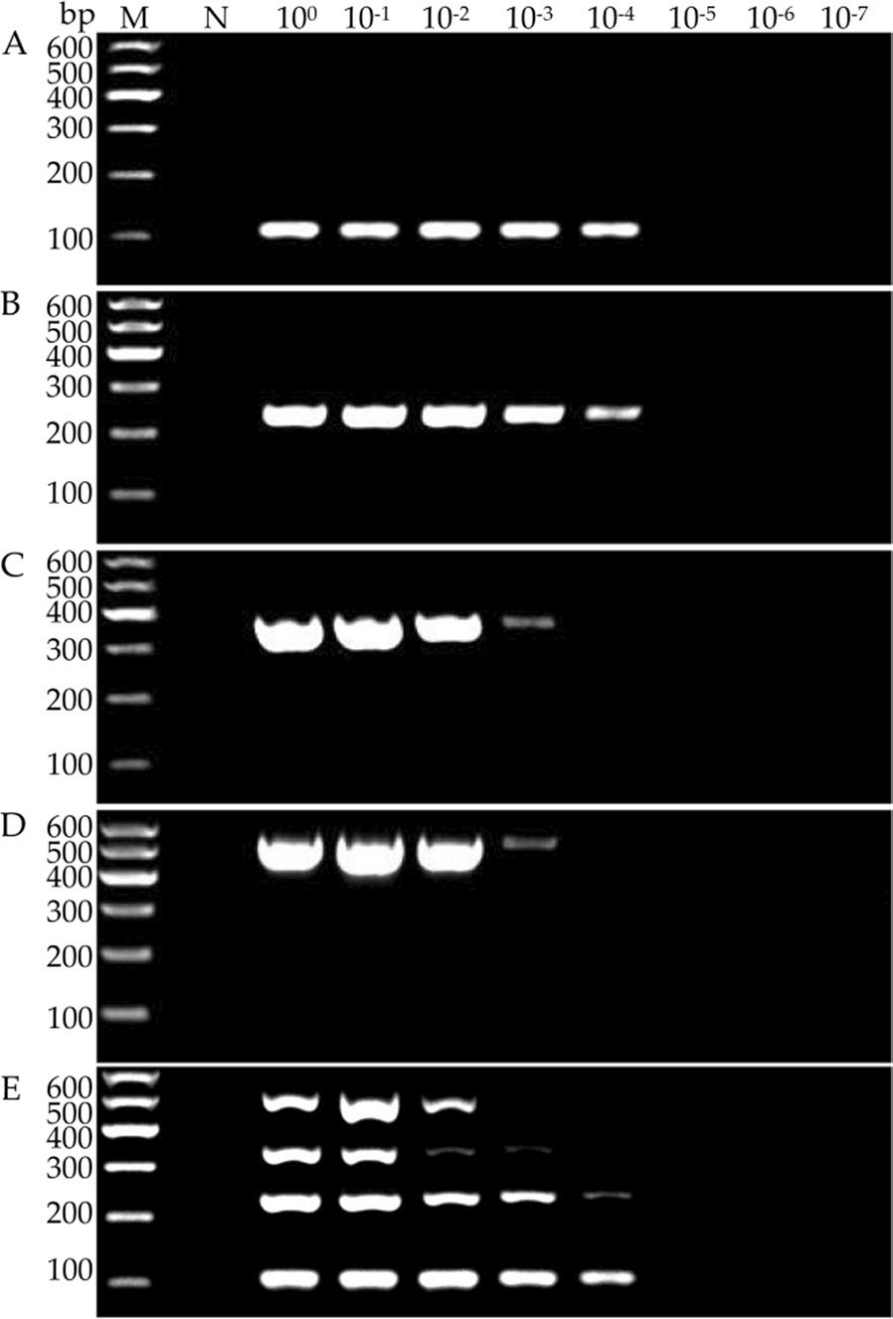
Comparison of sensitivities of uniplex RT-PCR assays for detection of LSV (**A**), LMoV (**B**), CMV (**C**), and PlAMV (**D**) and multiplex RT-PCR assay (**E**). Lane M, 100 bp plus DNA ladder; Lane N, negative control; Lanes10^0^–10^−7^, ten-fold serial dilutions of cDNA from a lily sample co-infected with four viruses

The sensitivity of each assay was determined using 10-fold serial dilutions (10^0^–10^–7^) of cDNA from lily samples co-infected with the four viruses. Both uniplex RT-PCR and multiplex RT-PCR could detect LSV and LMoV from cDNA diluted up to 10^–4^, and CMV from cDNA diluted up to 10^–3^. Uniplex RT-PCR and multiplex RT-PCR could detect PlAMV from cDNA diluted up to 10^−3^ and 10^–2^, respectively (Fig. 4).

### Application of multiplex RT-PCR assay in survey of lily viruses

The results showed that 146 of the 180 samples were infected with at least one virus (Additional file 2: Table S1). LSV were detected in 70 samples (38.89%), LMoV were detected in 67 samples (37.22%), CMV were detected in 97 samples (53.89%), and PlAMV were detected in 16 (8.89%), respectively (Table 2). Single infection was detected in 70 (38.89%) of the samples, and mixed infection was detected in 76 (42.22%) of the samples (Additional file 2: Table S1). To validate the accuracy of the multiplex RT-PCR survey, uniplex RT-PCR assays were performed to detect LSV, LMoV, CMV, and PlAMV separately from all collected samples, and the results were consistent with those of the multiplex RT-PCR (data not shown).

## Discussion

Designing and selecting proper primer combinations is of vital importance for establishing an efficient multiplex RT-PCR detection system [23]. In this study, virus-specific primers were designed based on the highly conserved regions of each virus. Using the final primer combinations, distinguishable amplicons with specific expected sizes for LSV (116 bp), LMoV (247 bp), CMV (359 bp, and PlAMV (525 bp) were obtained. The four products could be easily and clearly differentiated after electrophoresis on a 2% agarose gel since their size differences were 100 bp or more, and there were no non-specific products. Using a low concentration of agarose in the gel reduces the sensitivity of multiplex RT-PCR assays. Therefore, the agarose concentration in the gel was 2% in this study, which permitted the clear separation of the four amplicons.

Since the presence of more than one pair of primers in the same reaction mix may limit the sensitivity and compatibility of multiplex RT-PCR detection system, it is essential to optimize the PCR conditions to achieve an efficient multiplex detection. Here, annealing temperatures and cycling conditions were selected for optimization. Results showed that the intensity of the four target bands was the strongest at 53.8 °C and no further increase in intensity was achieved beyond 35 cycles (Fig. 2, 3), therefore, the optimal reaction annealing temperature and cycle number were set at 53.8 °C and 35, respectively. Sensitivity is another criterion to evaluate the multiplex system. The sensitivity analysis showed that the detection limits of the multiplex RT-PCR assay for the four viruses were similar to that of each uniplex RT-PCR assay, with only a slight reduction of 10-fold in the sensitivity for PlAMV detection in the multiplex assay (Fig. 4). The same results were also observed in other studies [23, 24]. This discrepancy may be partly due to competition in multiplex RT-PCR assay for key reactants, such as Taq polymerase and dNTPs.

Viral infection is one of the important diseases in lily cultivation [2]. In this study, 180 lily samples from different regions in China were tested using the developed multiplex RT-PCR assay. The results showed 146 samples were infected by at least one of the viruses, and the coinfection rate was 42.22% (Additional file 2: Table S1). Notably, 2/30 (6.67%) edible lily (*L. lancifolium* Thunb) samples were infected with PlAMV (Table 2). This is the first report of PlAMV in edible lily (*Lilium lancifolium* Thunb.) in China, which was only previously reported in ornamental lilies in China [25]. The high viral incidence could be due to the use of virus-infected bulbs for propagation and the lack of practical virus control and management measures. To manage these lily viral diseases, it is relevant to use resistant tolerant cultivars or virus-free propagation materials and use effective and sensitive detection methods to monitor the virus occurrence. The present developed multiplex RT-PCR assay will no doubt become an effective tool for solving these problems.

## Conclusions

In this study, by selecting proper primer combinations, annealing temperature, and cycle number, an effective multiplex RT-PCR assay was established for simultaneous detection of these four common viruses in lilies in a single reaction system. This assay will be useful for routine molecular diagnosis and epidemiological studies of these viruses.

## Supplementary Information

**Additional file 1: Figure S1.** Specific primers designed based on multiple sequences alignment for LSV. **Figure S2.** Specific primers designed based on multiple sequences alignment for LMoV. **Figure S3.** Specific primers designed based on multiple sequences alignment for CMV. **Figure S4.** Specific primers designed based on multiple sequences alignment for PlAMV

**Additional file 2: Table S1.** Survery of viral infection in lily plants collected from different geographic regions of China using the multiplex RT-PCR assay.

## Data Availability

The datasets generated and/or analyzed during the current study are not publicly available but are available from the corresponding author on reasonable request.
