# Supplementary material for: Development of a multiplex RT-PCR assay for simultaneous detection of Lily symptomless virus, Lily mottle virus, Cucumber mosaic virus, and Plantago asiatica mosaic virus in Lilies
Source: Virol J. 2022 Dec 16;19:219. doi: 10.1186/s12985-022-01947-3 (PMC9758769; doi:10.1186/s12985-022-01947-3)
Supplement: Supplementary file 1 — Additional file 1: Figure S1. Specific primers designed based on multiple sequences alignment for LSV. Figure S2. Specific primers designed based on multiple sequences alignment for LMoV. Figure S3. Specific primers designed based on multiple sequences alignment for CMV. Figure S4. Specific primers designed based on multiple sequences alignment for PlAMV [file 12985_2022_1947_MOESM1_ESM.docx]

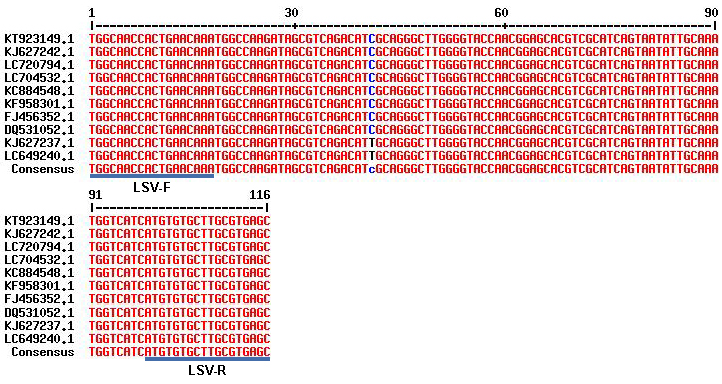


Fig. S1 Specific primers designed based on multiple sequences alignment for LSV


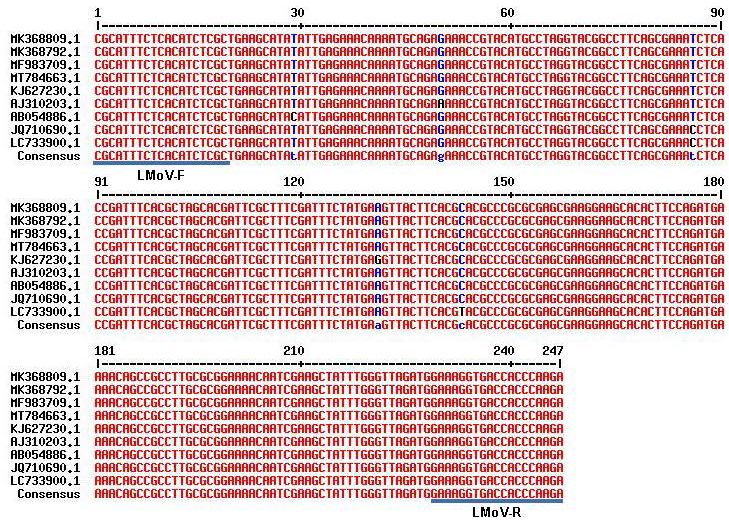


Fig. S2 Specific primers designed based on multiple sequences alignment for LMoV


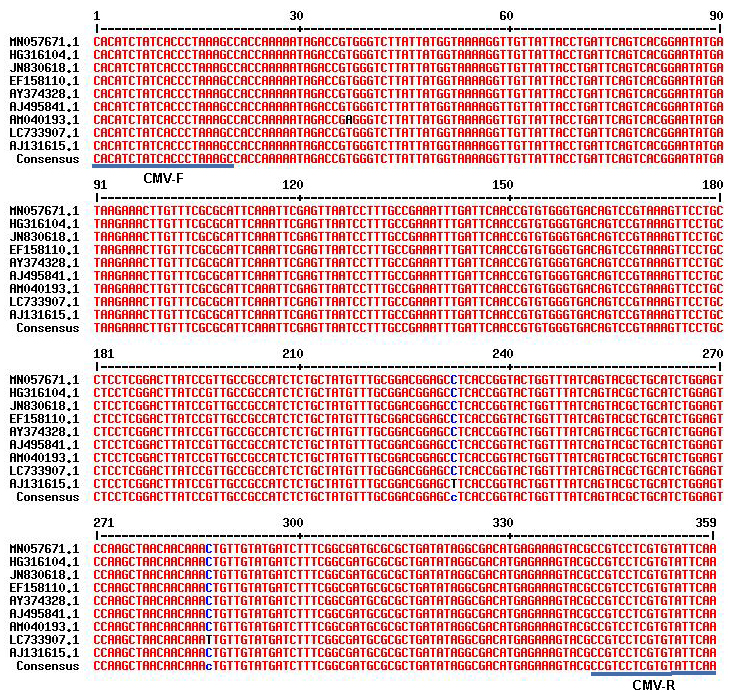


Fig. S3 Specific primers designed based on multiple sequences alignment for CMV


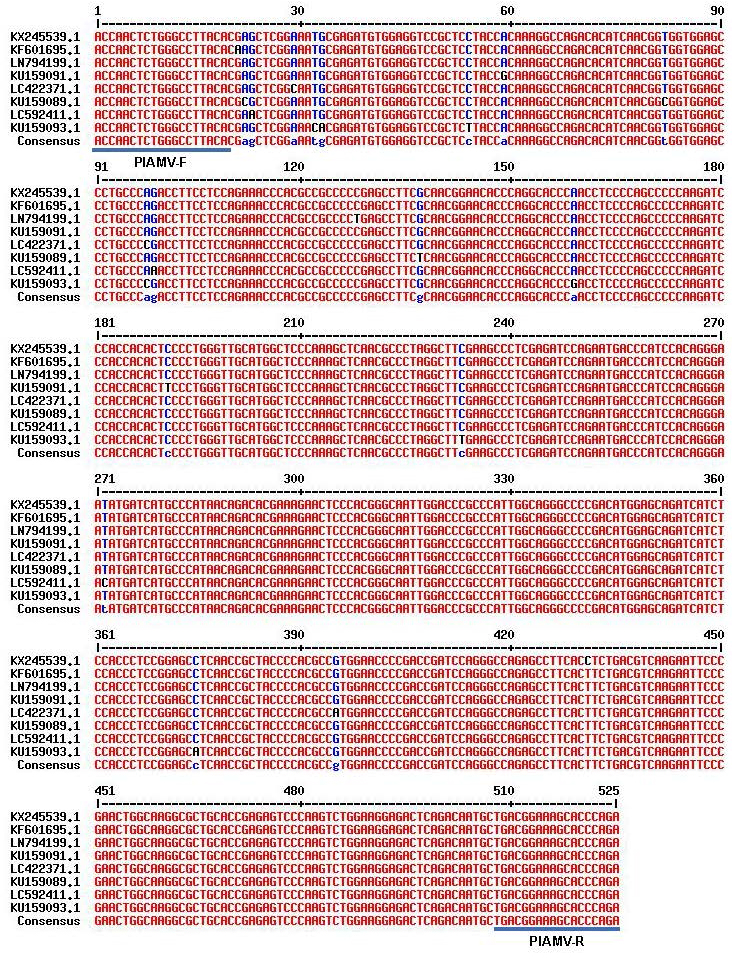


Fig. S4 Specific primers designed based on multiple sequences alignment for PlAMV
